# Free Radical Mediated Oxidative Degradation of Carotenes and Xanthophylls

**DOI:** 10.3390/molecules25051038

**Published:** 2020-02-26

**Authors:** Raphael C. Mordi, Olabisi T. Ademosun, Christiana O. Ajanaku, Ifedolapo O. Olanrewaju, John C. Walton

**Affiliations:** 1 Department of Chemistry, Chrisland University, Ajebo Road, Abeokuta, Ogun State 110222, Nigeria; 2 Department of Chemistry, Covenant University, Canaan Land, Km. 10, Idiroko Road, Ota, Ogun State 112242, Nigeria; olabisi.ademosun@covenantuniversity.edu.ng (O.T.A.); oluwatoyin.ajanaku@covenantuniversity.edu.ng (C.O.A.); ifedolapo.olanrewaju@covenantuniversity.edu.ng (I.O.O.); 3 EaStCHEM School of Chemistry, University of St. Andrews, St. Andrews, Fife KY16 9ST, UK

**Keywords:** free radicals, carotenoids, xanthophylls, oxidation, β-carotene, canthaxanthin, β-ionone, retinal, apo-carotenoids

## Abstract

This article reviews the excited-state quenching, pro-vitamin A activity and anticarcinogenicity of carotenes and xanthophylls in relation to their chemical structures. Excited-state quenching improved with the length of the conjugated chain structure. Pro-vitamin A activity was dependent on the presence of at least one beta-ionyl ring structure. The effectiveness of carotenoids as antioxidants depended on their ability to trap peroxyl radicals with production of resonance-stabilized carotenyl radicals. The products identified from oxidations of carotenes and xanthophylls with molecular oxygen and other oxidizing agents are presented. The free radical-mediated mechanisms that have been proposed to account for the different classes of products are reviewed.

## 1. Introduction

Carotenoids are yellow, orange, or red pigments which are widely distributed in the plant and animal kingdoms as well as among algae and other microorganisms. They are the major colorants of certain yellow, orange, or red flowers, vegetables, berries, mushrooms, insects, bacteria, feathers, and egg yolk [1]. In plants they are found in leaves together with chlorophyll, and in animals they are dissolved in fats or combined with protein in the aqueous phase. Ordinarily they are fat soluble. They are important constituents of most foods and act as coloring agents, additives, or stabilizers. They have also found application in the pharmaceutical industry as coating agents for drugs in order to give appetizing colors and flavors [2].

**Figure 1:**
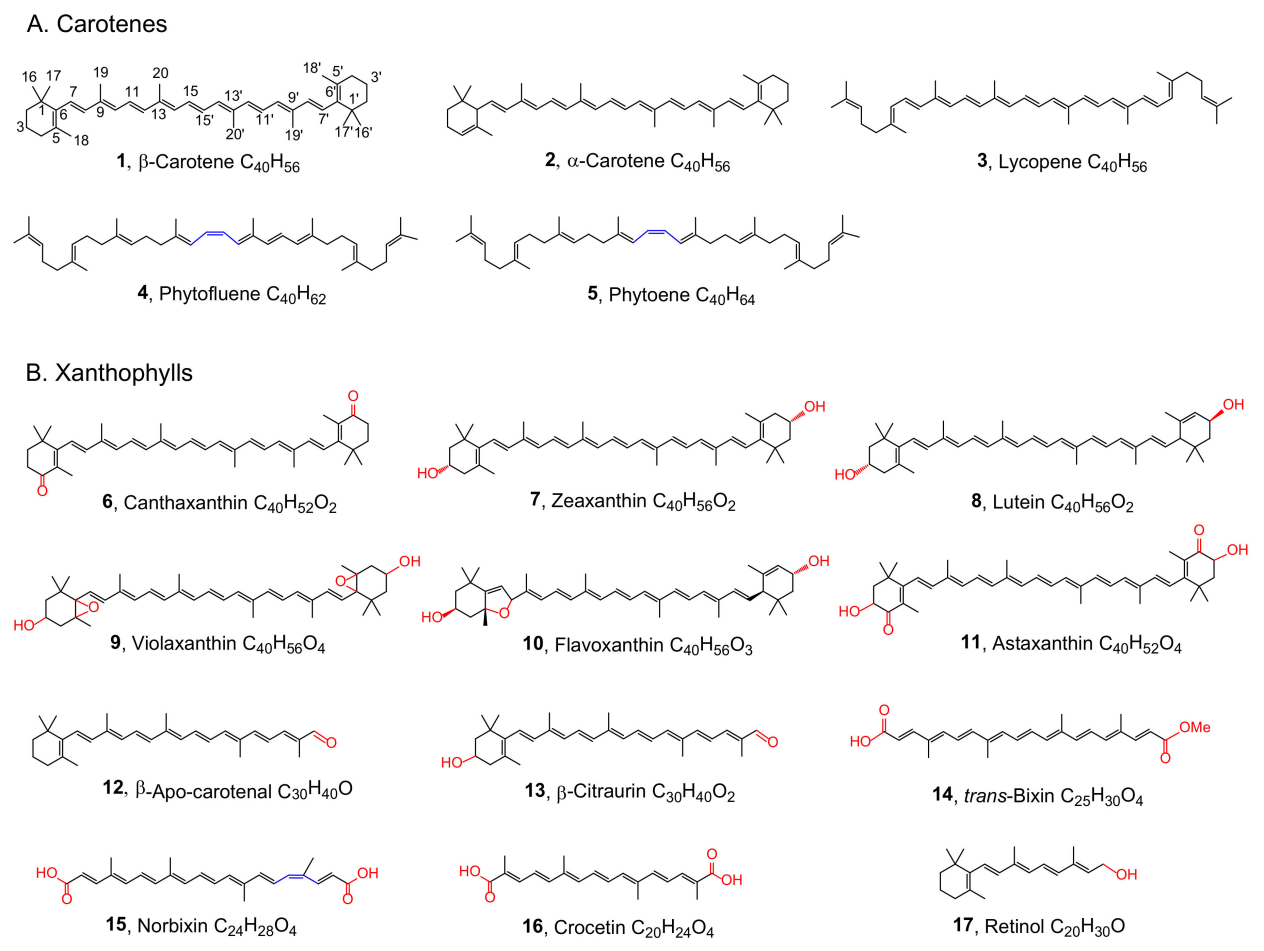
Structures of selected carotenes and xanthophylls.

The majority of naturally occurring carotenoids are tetraterpenoids made of eight isoprenoid units which arise through the ‘tail-to-tail’ condensation of two identical C_20_ units. Most carotenoids have a C_40_ carbon skeleton and possess a C_22_ central unit consisting of nine conjugated double bonds and four side-chain methyl groups. The end groups may or may not be cyclized, and the cyclic nature of the end group can be the same or different. A typical cyclic carotenoid contains two β-ionyl ring residues in its molecule, the archetype being β-carotene (**1**). Some carotenoids are known whose carbon skeletons possess fewer than 40 carbon atoms, for example β-citraurin (**13**), norbixin (**14**), and crocetin (**16**); a few with longer conjugated chains are also recognized. X-ray and spectral studies have shown that the double bonds usually have trans configurations but there are some exceptions (see Figure 1).

The two main classes of carotenoids are carotenes, which are purely hydrocarbons, and xanthophylls, which contain oxygen. The oxygenated substituents are usually hydroxy, epoxy, carboxy, or carbonyl groups. Most of the oxygen substituents appear on β-ionyl ring positions, although 5,8-epoxides (dihydrofurans) are known, such as flavoxanthin (**10**), as are apo-carbonyls, such as β-citraurin (**13**) and retinal. Some representative examples of each group are displayed in Figure 1.

Natural and laboratory roles of carotenoids are frequently protective, such as their use as agents for screening from light, their antioxidant activity, and their pro-vitamin A activity. Most of these roles lead to the degradative breakdown of their conjugated frameworks with the formation of suites of oxidized products. In some situations, particular oxidized products may continue, or actually be the principal agents in, the protective action. This article focusses on non-enzymatic roles with particular emphasis on carotenoid behavior with reactive oxygen species. The intention is, as far as possible, to shed light on the outcomes of radical-mediated processes and the mechanisms by which they operate.

## 2. Excited-State Quenching, Antioxidant, and Pro-Vitamin A Activity of Carotenoids

**Figure 2:**
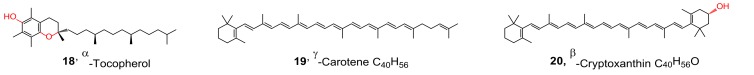
Structures of carotenoids and antioxidants.

The chemical structures of carotenoids suit them for several of their functions. It was originally thought that carotenes and xanthophylls served as light filters for chlorophyll and/or functioned as enzyme protectors in cells. Some other of the well-established functions of carotenoids are their pro-vitamin A activity, their antioxidant activity, their ability to quench singlet oxygen and their ability to inhibit the growth of certain tumors. In animal nutrition, one requirement for pro-vitamin A activity is the availability of at least one β-ionyl ring at one end of the molecule. Of all the known carotenoids, β-carotene possesses the highest pro-vitamin A activity due to its two retinyl units. In living systems enzymes convert it as needed to retinal. The biopotency of the other pro-vitamin A carotenoids is about half that of β-carotene, and that for the *cis*‒*trans* stereoisomers of β-carotene is lower than that for all-*trans* β-carotene. α-Carotene (**2**), γ-carotene, and the xanthophyll β-cryptoxanthin (**20**) have only single β-ionyl rings, so their vitamin A activity is lower (see Figure 2). Lycopene (**3**) and most other carotenoids lack β-ionyl rings and so have no vitamin A activity. They may, however, have other antioxidant properties.

That β-carotene serves as a source of vitamin A in animal nutrition has been known for a long time [3]. β-Carotene’s aptitude as a pro-vitamin A compound was tested by several experiments in which vitamin A-deficient rats were treated with it. These experiments resulted in the detection of retinol (**17**) in the intestinal walls of the animals [4,5,6]. It was during these experiments that the enzyme β-carotene-15,15′-dioxygenase was detected [7]. This enzyme cleaved β-carotene at its central bond with the production of retinal (**30**), which could then be reduced to retinol. However, in vitro experiments failed to detect retinal in some systems prepared from rat intestines and were, therefore, unable to demonstrate quantitatively the conversion of β-carotene to retinol in vivo. Other reports have appeared that suggest protection, during lycopene and β-carotene consumption, against the menace of cardiovascular diseases including atherosclerosis, myocardial infarction, and stroke [8,9,10].

In the absence of antioxidants, carotenoids are unstable to heat, light and air/oxygen, degrading to compounds of smaller molecular weight. Following a report that β-carotene might act as an anticarcinogenic agent [11], several research groups began working to establish the effectiveness of these compounds as antitumor agents and also to determine the mechanism by which they function. The important question is whether it is the carotenoid or its degradation products that provide the protection and by what mechanism they act. Recent research activities through the characterization of their degradation products point toward a mechanism involving free radicals.

A mechanism was proposed for the metabolism of β-carotene that involved degradation from one end of the molecule, thus forming a series of apo carotenals. These could either undergo additional metabolism with formation of retinal (**30**) or retinoic acid or were oxidized to their respective acids and then metabolized to retinoic acid [12]. Evidence in support of the formation of these apo-carotenals during in-vitro degradations by the tissue extracts of rats, ferrets, monkeys, and humans has been presented [13,14,15].

Some carotenoids are also known to protect cells against the harmful effects of light, oxygen, and photosensitizing agents. Many have the capacity to quench or inactivate the excited states of molecules. This process is best exemplified by the quenching of those states formed in photosensitized reactions. The quenching capability depends on the length and rigidity of the molecule, the length of the conjugated chromophore, the nature of the end groups—whether cyclic or acyclic—and on the presence of substituents. All these structural features affect the light-absorbing and quenching ability. Foote and Denny showed that carotenoids could quench excited singlet oxygen and that the process was limited by the number of double bonds in the molecule [16]. The maximum quenching effect was found to be offered by molecules that had nine or more double bonds and this helped to redefine the role of carotenoids as protective agents. They proposed that the carotenoid directly interacts with singlet oxygen at a low concentration to give a triplet-state carotenoid and triplet ground-state oxygen. They concluded that the quenching was mainly the transfer of excitation energy, and not an oxidation of the carotenoid pigment [16,17,18].

Mathews-Roth et al. also demonstrated that carotenoids containing nine or more conjugated double bonds were protective against photosensitization, whereas little protection was seen with those containing seven or fewer such bonds [19]. They observed that carotenoids containing nine conjugated double bonds were not as effective as lutein (**8**) or β-carotene, which contain ten and eleven conjugated double bonds, respectively. A small level of protection was observed with ζ-carotene, which contains seven conjugated double bonds, but phytofluene (**4**) and phytoene (**5**), in which the conjugation extends to only five and three conjugated double bonds, respectively, offered no protection [20,21,22,23,24]. Additional research concurred in showing that carotenoids with eleven, ten, nine, and seven conjugated double bonds were effective in inhibiting photoreduction reactions, but phytoene (**5**) was not. The photochemistry and mechanism of action have been reviewed [25,26].

β-Carotene has been reported to quench or inhibit directly radical species and lipid peroxidation in liposomes, thereby acting as an antioxidant by protecting cells and organisms from oxidative damage [21,27]. For example, peroxyl radicals produced by exposing carbon tetrachloride to pulse radiolysis in the presence of oxygen (CCl_3_OO^•^ radicals) reacted with β-carotene.

In their studies on the antioxidant activity of carotenoids toward lipid peroxidation, Burton and Ingold showed that β-carotene belongs to a previously unknown class of biological antioxidants. These compounds were especially effective at trapping radicals at low oxygen partial pressures, such as those found in most tissues under physiological conditions [28]. They suggested that, unlike the chain-breaking antioxidants such as tocopherols (which trap by donating a hydrogen atom), β-carotene exerts its antioxidant activity by a mechanism in which the chain-propagating peroxyl radical is trapped by addition to the conjugated polyene system of the carotene. The resulting carbon-centered radical, which is resonance stabilized, predominates over the chain-carrying peroxyl radical at low oxygen partial pressure. These results may explain β-carotene’s effectiveness at inhibiting lipid peroxidation, as peroxyl radicals are considered to be the propagating species in this reaction. The overall effect is to divert a potentially damaging lipid chain reaction into a much less harmful side reaction involving the considerably more expendable β-carotene.

Carotenoids’ quenching ability against singlet oxygen and free radical scavenging, as well as their antioxidant activity, have been shown to be dependent on their substitution. For example, astaxanthin (**11**) with two carbonyl and two hydroxyl groups was found to be about ten times more effective than zeaxanthin (**7**), lutein (**8**), tunaxanthin, canthaxanthin (**6**), and β-carotene, all with fewer substituents, and 100 times as effective as α-tocopherol (**18**) at inhibiting lipid peroxidation mediated by active forms of oxygen (see Figure 2). The antioxidant activities of canthaxanthin (**6)** and astaxanthin (**11**) were found to last longer than those of β-carotene and zeaxanthin (**7**). This led to the suggestion that substituting an oxo group at the 4(4′) position increases the efficiency of a carotenoid’s peroxyl radical-trapping ability. This is because the electron-withdrawing ability of the oxygen atoms substantially reduces the unpaired electron density on the carbon skeleton decreasing the reactivity of the carbon-centered radical toward molecular oxygen [29,30].

It was found that the initial oxidation rate increased during the AIBN- (2,2′-azobis(2-methylpropionitrile)-initiated oxidation of methyl linoleate in the presence of β-carotene. Faria and Mukai also reported increased rates of oxygen uptake during the irradiation of linoleic acid in the presence of β-carotene [31]. Matsushita and Terao [20] found that in the absence of tocopherols, β-carotene was rapidly autoxidized during irradiation. The ability of α-tocopherol (**18**) to protect β-carotene from autoxidation was recently reported and that the protection was dose dependent at sufficiently high concentrations of α-tocopherol [32]. Based on their results, Terao et al. suggested that the autoxidation products of β-carotene have the capacity to induce an autocatalytic oxidation of the oils studied. Earlier, Burton and Ingold had suggested that, because β-carotene does not have the same structural features commonly associated with chain-breaking antioxidants, the extensive system of conjugated double bonds in the molecule imparts prooxidant character [28].

## 3. Carotenoids and Carcinogenesis

Carotenoids have been added to the diets of experimental animals in an attempt to inhibit carcinogenesis induced by UV light, chemical carcinogens, or a combination of both [33,34,35]. Results from these studies revealed that β-carotene significantly reduced the incidence of skin tumors and lowered the number of other tumors formed in these animals. Mathews-Roth et al. showed how β-carotene ingestion affects the photosensitivity associated with erythropoietic protoporphyria (EPP) [36]. EPP is a genetic disease of porphyrin metabolism, in which protoporphyrin is the photosensitizer. This causes a burning sensation in the skin followed by varying degrees of redness and swelling. They showed that patients taking β-carotene were able to tolerate exposure to sunlight without developing the symptoms associated with EPP and determined the β-carotene dosage that was satisfactory for the treatment of various age groups. β-Carotene has been shown to protect against lung cancer and probably against stomach cancer, and it may also inhibit cancer of the ovary, cervix, breast, and other cancers but not those of the colon or rectum [37].

An effect of β-carotene on children with cystic fibrosis has also been demonstrated [38]. It was observed that β-carotene levels were low at different stages, irrespective of the severity of the disease, despite normal levels of vitamin E. Consequently, significant lipid peroxidation took place because of increased free radical activity.

The anticarcinogenicity of β-carotene may reflect its vitamin A activity, but only in specific tissues and may be, at least partly, attributed to its antioxidant effect in so far as oxygen radicals are implicated in processes leading to human cancers [39]. However, the affects attributed to carotenoids are because of an intrinsic property of the molecules themselves and not a result of their pro-vitamin A activity. This was discovered when canthaxanthin (**6**) and phytoene (**5**), which cannot be converted to retinal (**30**) and, therefore, are considered non-pro-vitamin A carotenoids, were found to be effective in cellular and animal studies [40]. The results from these studies show that, irrespective of their vitamin A activity, carotenoids can prevent or slow the growth of skin tumors induced by UVB or chemical carcinogens. The mechanism for this action has yet to be understood, but studies suggest that β-carotene is effective at both the initiation and promotion stages of carcinogenesis, while other studies indicated that it may be effective at either the initiation, promotion, or during the subsequent development of tumors [41,42]. Evidently, the anticarcinogenicity does not depend on the presence of a β-ionyl ring but may be contingent on the chain of conjugated bonds and its extent.

## 4. Oxidative Degradation of Carotenoids

Carotenoids are stable in biological systems because they form protein complexes in their natural environment and because of the presence of natural antioxidants. However, their stabilities in plants vary widely. The pathways for their degradation have not yet been elucidated, because different carotenes have stabilities that differ under different physiological conditions. In isolated or pure form, carotenoids in the presence of oxygen and light degrade rapidly. Their ability to interact with free radicals, including peroxyl radicals, results from their systems of conjugated double bonds, which render them open to oxidation. The likelihood of them undergoing autoxidation also depends on the number and type of substituents in their β-ionyl ring residues. The relative oxidative susceptibility was related to their polarity, for example, in the order β-carotene (**1**) > β-apo-8′-carotenal > cryptoxanthin (**20**) > canthaxanthin (**6**) > zeaxanthin (**7**) [43].

It was proposed that adding two water molecules at the central double bond of β-carotene should give rise to two molecules of vitamin A [44,45]. As mentioned earlier, in vitro experiments failed to show the quantitative conversion of β-carotene to vitamin A and it was also shown that vitamin A was twice as active as β-carotene. However, the chemical oxidation of β-carotene by treatment with hydrogen peroxide and glacial acetic acid in the presence of a catalyst (OsO_4_) afforded only small amounts of retinal (**30**) [46]. When the reaction’s progress was followed, with time, a series of β-apo-carotenals was isolated [47]. Following these observations, it was suggested, based on physicochemical considerations, that the central 15,15′-double bond should be more stable than the terminal ones because of resonance, implying that terminal attack should be preferable to central fission [48]. It was subsequently found that the electron density is greatest at the terminal double bonds of the β-carotene molecule [49].

Two theories were proposed in the 60s and 80s regarding the oxidative degradation mechanism [12,50]. One was the random cleavage theory in which attack occurs randomly at different sites of the β-carotene double bond chain. The other was the central cleavage theory in which attack is at the central double bond of the molecule. In order to account for the fact that β-carotene was half as active as retinol, Glover proposed a “β-oxidation mechanism”, which involved attack and cleavage at the terminal double bonds of the conjugated system [12]. This cleavage continued with the successive removal of two carbon units until the 15,15′-double bond was reached, when further β-oxidation was blocked by the methyl group located on C(**13**), which is at the β-position with respect to the central double bond of the β-carotene molecule.

During our studies, retinal (**30**) was subjected to self-initiated oxidation at 30 °C in benzene and we found that after more than 220 h on stream, less than 10 per cent of the retinal had reacted [51]. We suggested that there must be a limiting chain length for rapid autoxidation, which is not inconsistent with Glover’s β-oxidation mechanism. The recent isolation of apo-carotenals and retinal from the intestines of rat, human, monkey, ferret, and chicken tissues has been interpreted on the basis of non-specific attack on any one of the double bonds of the β-carotene molecule [14,15,50].

## 5. Products from Oxidations of Carotenes and Xanthophylls

The oxidations of carotenes and xanthophylls by molecular oxygen and other oxidizing agents have been examined under various conditions and the products are basically similar, although the proportions vary depending on temperature, oxygen pressure, light, matrix composition etc. The enzymatic oxidation of β-carotene with rat liver homogenates was reported to produce retinal that was subsequently reduced to retinol (**17**) [7]. Tang et al. isolated β-apo-13-carotenone and β-apo-14′-carotenal from the treatment of β-carotene with various enzymatic homogenates [14]. Further work with tissue homogenates enabled β-apo-12′-, -10′-, and -8′-carotenals, retinal, and retinoic acid to be identified [15].

**Scheme 1:**
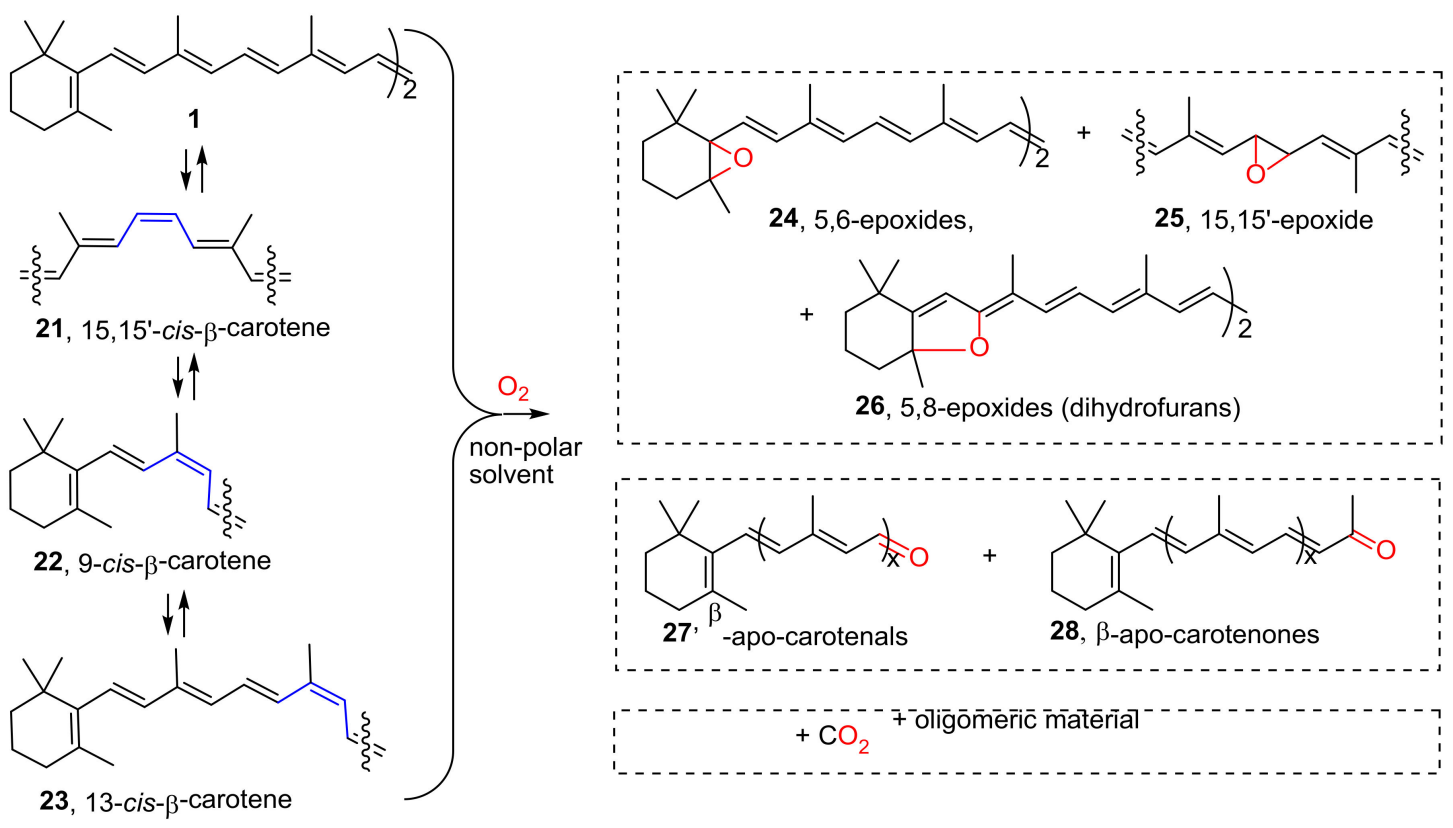
Product types from the self-initiated oxidation of carotenes by molecular oxygen.

El-Tinay and Chichester found that the oxidation of β-carotene with molecular oxygen in toluene afforded β-carotene-5,6-monoepoxide (**24**) and the corresponding 5,8-monoepoxide (**26**) together with diepoxides [49]. For β-carotene oxidation by molecular oxygen in non-polar solvents we found the initial products included the 5,6-epoxides, 5,8-epoxides (**24**, **26**), the 15,15′-epoxide (**25**), a series of β-apo-carotenals (**27**), and β-apo-carotenones (**28**) together with carbon dioxide and oligomeric material (Scheme 1). At long oxidation times the epoxides became less important, small amounts of alcohols and probably acids were also formed, and the shorter chain carbonyl components tended to predominate [51,52]. In addition, product spectra gave evidence that isomerization of *trans*-**1** took place with the production of *cis* isomers of β-carotene. Krinsky et al. demonstrated the formation of a homologous series of carbonyl products, including β-apo-13-carotene, retinal, β-apo-14′-carotenal, β-apo-12′-carotenal, and β-apo-10′-carotenal during the autoxidation of β-carotene [32]. Kennedy and Liebler identified 5,6-epoxy- (**24**) and 15,15′-epoxy-β-carotenes (**25**) from the treatment of β-carotene with peroxyl radicals [53]. Rodriguez and Rodriguez-Amaya studied the oxidation with mCPBA (*meta*-chloroperbenzoic acid) and observed similar epoxides and diepoxides, whereas oxidation with KMNO_4_ afforded mainly the carotenals and carotenones [54].

The first observations of *trans* to *cis* isomerization of **1** were by Friend [55] from the study of the β-carotene UV band at 300 nm during oxidation. We confirmed this process and showed that isomerization of the carbonyl oxidation products also took place [51]. *Trans* to *cis* isomerization of carotenes has been studied by several other research groups. The formation of *cis* isomers was implied from the work of Doering and Sarma [56] with model, semi-rigid, conjugated systems of three, five, and seven double bonds. The identities of the *cis* isomers from β-carotene have been confirmed by other workers as 9-*cis*- (**22**) and 13-*cis*-β-carotene (**23**) [57,58,59,60,61].

**Scheme 2:**
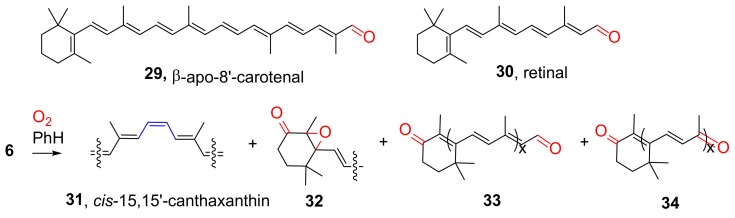
Xanthophylls and their oxidation products.

Xanthophyll oxidation products have also been studied but to a lesser extent. The self-initiated oxidation of β-apo-8′-carotenal (**29**) in benzene solution took place more slowly than β-carotene but gave rise to a series of carbonyl compounds similar to that obtained from **1**, i.e., **24** to **26** [51]. The spectral data indicated that *cis* isomers of **29** were also formed and probably small amounts of the 5,6-epoxide. Retinal (**30**) was observed to be significantly more resistant to oxidation under the same conditions and only minor amounts of similar products were detected. The products from the oxidation of canthaxanthin **6** by molecular oxygen in solution have been studied in detail [62]. These included the isomer *cis*-15,15′-canthaxanthin (**31**) and minor amounts of 4-oxo-5,6-epoxides **32** (possibly also 4-oxo-5,8-epoxides) with a series of 4-oxo-carbonyls **33** and **34** as the main components (see Scheme 2).

Møller et al. investigated the oxidation of norbixin (**15**) in aqueous solution [63]. They observed, by mass spectrometry, a series of polyoxygenated norbixins together with another series of norbixin-derived carbonyl components analogous to those described above. The polyoxygenated norbixins were believed to include epoxides, diepoxides, and hydroperoxides.

β-Carotene and canthaxanthin both contain six branching methyl groups that have allylic H atoms. Allylic H atoms are readily able to be abstracted by free radicals because the resulting allyl radicals are resonance stabilized. It was interesting to note that no product from the attack at any of these six methyl groups was identified from the oxidations of these carotenoids. It can be concluded that free radical additions to the unsaturated chains are significantly more favorable than allylic H-atom abstractions.

## 6. Mechanisms of the Oxidation of Carotenes and Xanthophylls by Molecular Oxygen

The oxidation rate of β-carotene depends on the pressure of oxygen but was found to be rather insensitive to solvent. Even in methyl caprylate solvent, as a lipid model, the rate of oxygen uptake was about the same as in non-polar solvents such as benzene. The sigmoidal oxygen uptake curves suggested the process was autocatalytic. The reactions were accelerated by AIBN and other radical initiators and inhibited by radical scavengers such as BHT (butylated hydroxytoluene or 2,6-di-tert-butyl-4-methylphenol) and α-tocopherol. Consequently, these oxidations have been recognized as free radical-mediated processes since the earliest studies [27,49,51,64].

**Scheme 3:**
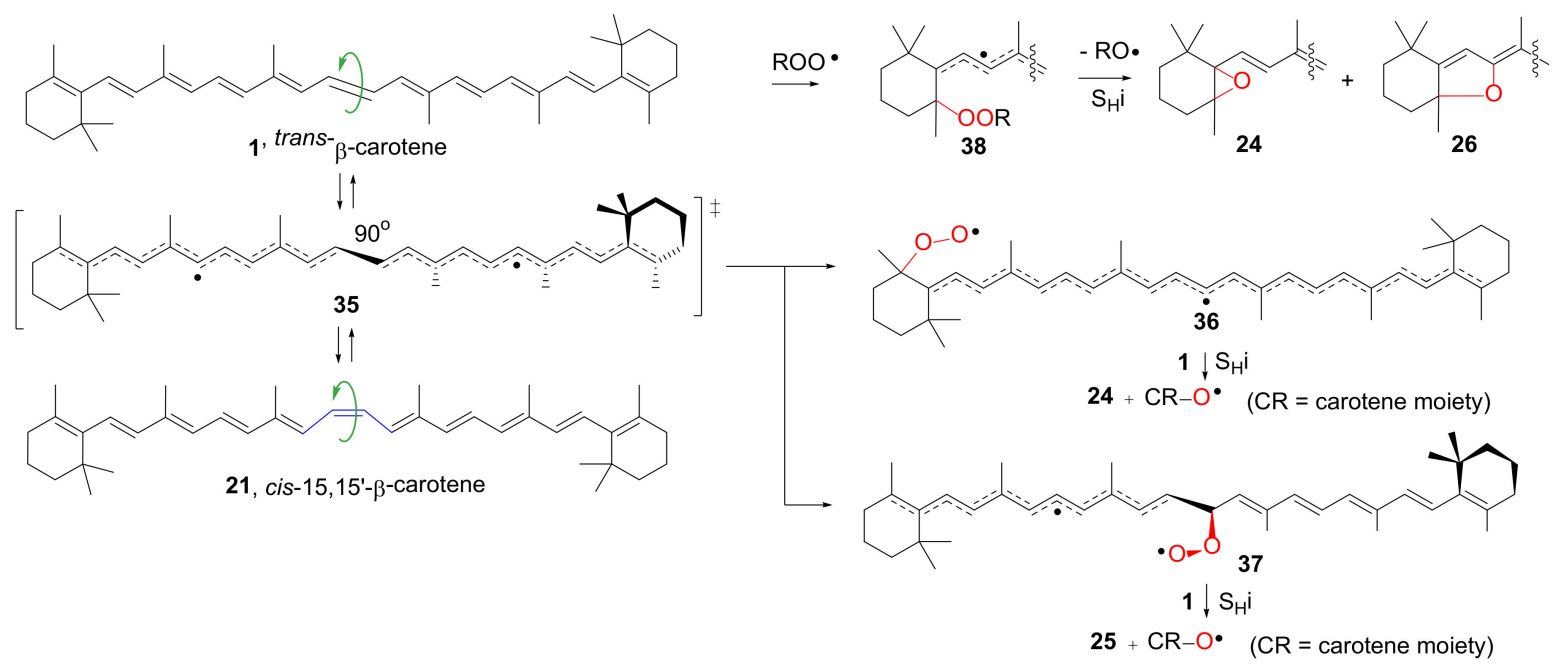
Initiation and epoxide formation.

The occurrence of *trans* to *cis* isomerization with carotenes and xanthophylls takes place from the earliest onset of oxidation. The barrier to internal torsion about the central 15,15′-C=C bond in β-carotene and the related carotenoids is lower than for isolated C=C double bonds. Internal rotation in the 15,15′-bond uncouples the bonding pair of π-electrons to produce a triplet diradical (**35**, see Scheme 3). The individual unpaired electrons of this are delocalized in the two halves of the conjugated system over 5 π-bonds each. This leads to very significant lowering of the energy of this transition-state species.

The coupling of diradical **35** with oxygen could take place at either end with production of peroxyl diradical **36**. Alternatively, oxygen could be picked up at sites along the chain, for example at C-15 or C-15′ to afford peroxyl diradicals such as **37**. The peroxyl radical **36** may add to another β-carotene molecule and the resulting peroxide would then undergo intramolecular homolytic substitution (S_H_i) with production of the 5,6-epoxy-β-carotene **24** plus a carotene alkoxyl radical CR-O^•^. In the same way intermediate **37** would yield 15,15′-epoxy-β-carotene **25** (see Scheme 3). There are numerous precedents for this kind of homolytic substitution [65,66,67,68]. A second, probably more important, route to epoxides accompanies this initiation process. The addition of a peroxyl radical to C-5 of the carotenoid, at the end of the conjugated chain, will produce an extensively stabilized intermediate radical **38**. The latter can then undergo S_H_i at C-6 or at C-8 with the production of 5,6-epoxide **24** and/or 5,8-epoxide **26** (Scheme 3). The attachment of ROO^•^ to terminal C-5 affords the most thermodynamically stabilized intermediate (**38**) and this probably accounts for the preponderance of 5,6- and 5,8-epoxides.

**Scheme 4:**
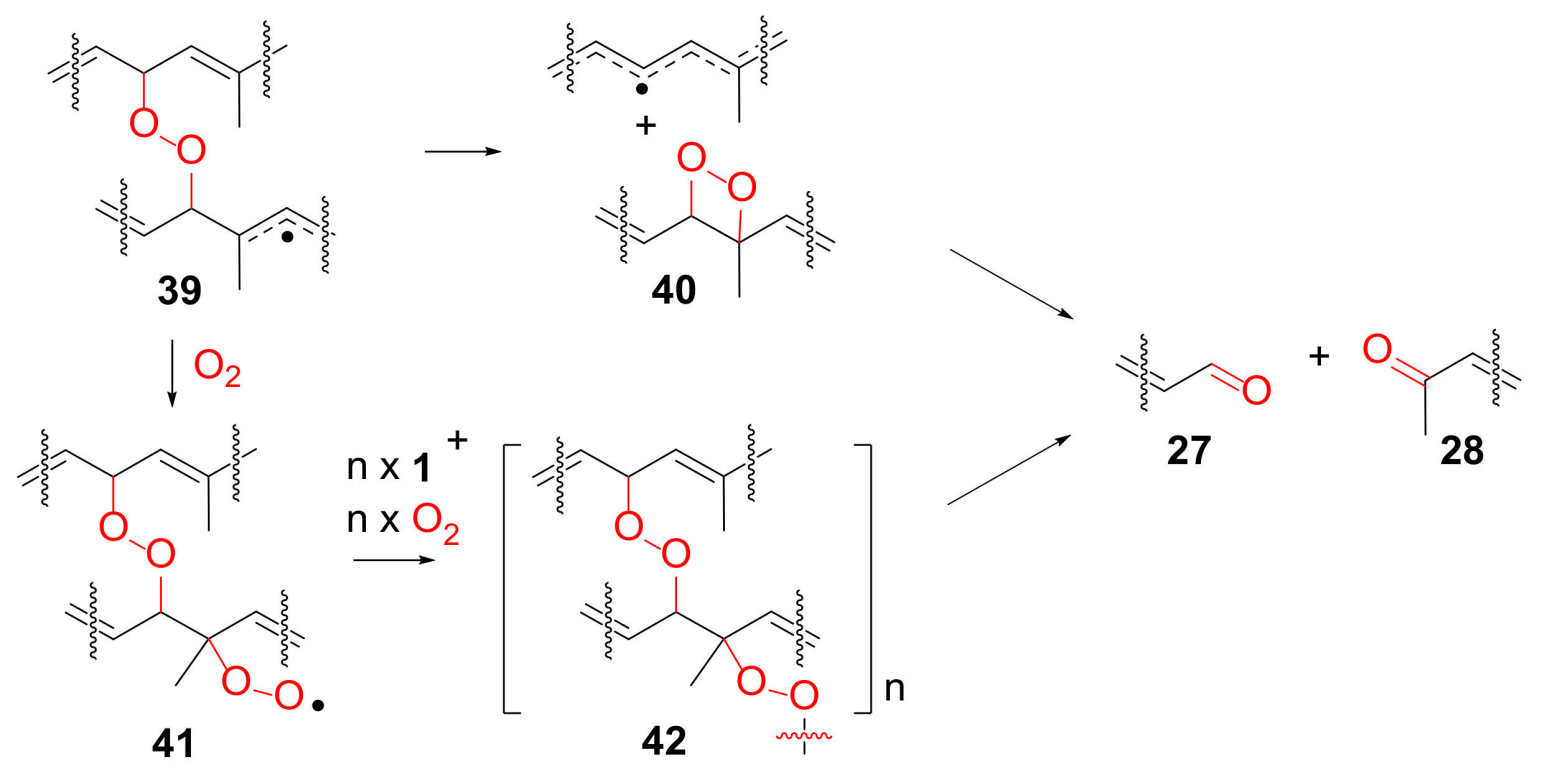
Alternative mechanisms for production of carbonyl components.

Series of β-apo-carotenals and β-apo-carotenones were important products in oxidations of both carotenes and xanthophylls. Several plausible routes to these have been suggested [14,15,51,62] and two are illustrated in Scheme 4.

The carotenoid peroxides **39** (analogous to **38**) could undergo an alternative S_H_i process that yields dioxetanes **40**. The resonance stabilization of the carotenyl radical that is produced at the same time would lower the free energy of this process. The unstable dioxetanes **40** would then dissociate rapidly to produce β-apo-carbonyls (**27** and **28**). Alternatively, a series of additions of lengthening carotenyl-peroxyl radicals could give rise to oligomeric peroxides **42**. The carbonyls could arise from the thermal decomposition of this oligomer as it “unzipped.” The oligomeric peroxyl radicals **41** are formed from the coupling of oxygen with the delocalized carotenoid radicals **39**. Could this process take place randomly at all the sites of unpaired electron density in the radical or would coupling be directed to a specific site or sites? Some light was shed on this question during an EPR (Electron Paramagnetic Resonance) spectroscopic study of spin trapping of the delocalized retinyl and β-ionyl radicals [69]. The spectra showed that nitroxide spin adducts were formed at all the secondary and tertiary C atoms of the carotenoid radical’s conjugated systems. There was a small preponderance of coupling at secondary rather than tertiary sites. Oxygen is smaller than the nitroso spin trap and more reactive, so it is probable that the coupling of radicals like **39** with oxygen takes place randomly at all possible sites.

The carbonyl components themselves oxidize as the reaction proceeds. The β-apo-carotenals are more susceptible than the β-apo-carotenones because of their labile acyl H atoms. Analysis showed that, as expected, the methyl ketones predominated over the aldehydes at long oxidation times. Carbonyl compounds derived from scission of every available double bond, except the ring double bond, were identified, in keeping with a very unselective oxygenation process.

The main difference in the spectrum of products from canthaxanthin oxidation was the comparative paucity of epoxides. Possibly, the presence of the carbonyl function at C-4 diverted intermediates analogous to **38** down alternative pathways.

## 7. Conclusions

Carotenoids can function as antimutagenic compounds, and animal studies support the idea that dietary carotenoids can prevent the appearance of skin tumors induced either by UV light or by a combination of chemical carcinogens and UV radiation. There is no certainty about which of the oxidized products are responsible for the anticancer activity. They may resemble the β-apo-carotenals and β-apo-carotenones that are non-toxic and are known to modulate retinoid receptors and to be active against cell proliferation, tumors, and tumorigenic viruses.

The mode of action of the carotenoids has been linked to their ability to interact with free radicals and peroxyl radicals and the stability of the resonance-stabilized carbon-centered carotenyl radicals formed. Although it has not been conclusively proved that retinal is formed directly from β-carotene by central cleavage, all available evidence so far points to the random cleavage of carotenoids to give apo-carotenoids as the main mechanism of degradation. Enzymatic oxidations of β-carotene by various tissue homogenates produced product series similar to those from self-initiated oxidations. This hints that the mechanisms may also be similarly mediated by peroxyl free radicals.

When canthaxanthin is used as a food additive for coloring or for medication, its effectiveness will be improved by formulation with radical inhibitors, such as tocopherols or other hindered phenols.

## References

[1] Rodriguez-Amaya D.B. (2019). Update on natural food pigments–A mini-review on carotenoids, anthocyanins, and betalains. Food Res. Int..

[2] Munzel K., Bauernfeind J.C. (1981). Carotenoids in pharmaceutical and cosmetic products. Carotenoids as colorants and vitamin A precursors.

[3] Moore T. (1930). Vitamin A and Carotene: The absence of the liver oil vitamin A from carotene. VI. The conversion of carotene to vitamin A in vivo. Biochem. J..

[4] Mattson F.H., Mehl J.W., Deuel H. (1947). Studies on carotenoid mechanism. 7. The site of conversion of carotene to vitamin A in the rat. Arch. Biochem..

[5] Glover J., Goodwin T.W., Morton R.A. (1948). Studies in Vitamin. 8. Conversion of β-carotene into vitamin A in the intestine of the rat. Biochem. J..

[6] Thompson S.Y., Ganguly J., Kon S.K. (1949). The conversion of β-carotene to vitamin A in the intestine. Br. J. Nutr..

[7] Olson J.A., Hayaishi O. (1965). The enzymatic conversion of β-carotene into vitamin A by soluble enzymes of rat liver and intestine. Proc. Natl. Acad. Sci. USA.

[8] Rissanen T.H., Voutilainen S., Nyyssonen K., Lakka T.A. (2001). Low serum lycopene concentration is associated with an excess incidence of acute coronary events and stroke: The Kuopio Ischaemic Heart Disease Risk Factor Study. Br. J. Nutr..

[9] Sesso H.D., Buring J.E., Norkus E.P., Gaziano J.M. (2004). Plasma lycopene, other carotenoids and retinol and the risk of cardiovascular disease in women. Am. J. Clin. Nutr..

[10] Voutilainen S., Nurmi T., Mursu J., Rissanen T.H. (2006). Carotenoids and cardiovascular health. Am. J. Clin. Nutr..

[11] Peto R., Doll R., Buckley J.D., Sporn M.B. (1981). Can dietary β-carotene materially reduce human cancer rates?. Nature.

[12] Glover J. (1960). The conversion of β-carotene to vitamin A. Vitamins and Hormones.

[13] Sharma R.V., Mathura S.N., Dmitrovskii A.A., Das R.S., Ganguly J. (1976). Studies on the metabolism of beta-carotene and apo-β-carotenoids in rats and chickens. Biochem. Biophys. Acta..

[14] Tang G., Wang X.D., Russell R.M., Krinsky N.I. (1991). Characterisation of β-Apo-13-carotenone and-Apo-14’-carotenal as enzymatic products excentric cleavage of β-carotene. Biochemistry..

[15] Wang X.D., Tang G., Fox J.G., Krinsky N.I., Russell R.M. (1991). Enzymatic conversion of β-carotene into -apo-carotenals and retinoids by human, monkey, ferret and rat tissues. Arch. Biochem. Biophys..

[16] Foote C.S., Denny R.W. (1968). Chemistry of singlet oxygen. VII. Quenching by beta-carotene. J. Am. Chem. Soc..

[17] Ronsein G.E., Miyamoto S., Bechara E., Di Mascio P., Martinez G.R. (2006). Oxidação de proteínas por oxigênio singlete: Mecanismos de dano, estratégias para detecção e implicações biológicas. Quím. Nova.

[18] Müller L., Caris-Veyrat C., Lowe G., Böhm V. (2016). Lycopene and its antioxidant role in the prevention of cardiovascular diseases-a critical review. Crit. Rev. Food Sci. Nutr..

[19] Mathews-Roth M.M., Wilson T., Fujimori E., Krinsky N.I. (1974). Carotenoid chromophore length and protection against photosentisation. Photochem. Photobiol..

[20] Matsushita S., Terao J., Simic M.G., Karel M. (1980). Singlet oxygen-initiated photooxidation of unsaturated fatty acid esters and inhibitory effects of tocopherols and β-carotene. Autoxidation in Food and Biological Systems.

[21] Packer J.E., Mahood J.S., Mora-Arellano V.O., Slater T.F., Wilson R.L., Wolfenden B.S. (1981). Free radicals and singlet oxygen scavengers: Reaction of a peroxyl-radical with β-carotene, diphenyl furan and 1,4-diazobicyclo(2,2,2)-octane. Biochem. Biophys. Res. Commun..

[22] Di Mascio P., Kaiser S., Sies H. (1989). Lycopene as the Most Efficient Biological Carotenoid Singlet Oxygen Quencher. Arch. Biochem. Biophys..

[23] Lee S.-H., Min D.B. (1990). Effects, Quenching Mechanisms, and Kinetics of Carotenoids in Chlorophyll-sensitized Photooxidation of Soybean Oil. J. Agric. Food Chem..

[24] Jung M.Y., Min D.B. (1991). Effects of Quenching Mechanisms of Carotenoids on the Photosensitized Oxidation of Soybean Oil. J. Am. Oil Chem. Soc..

[25] Krinsky N.I. (1979). Carotenoid protection against oxidation. Pure Appl. Chem..

[26] Krinsky N.I. (1989). β-Carotene: Functions, New Protective Roles for Selected Nutrients. Current Topics Nutrition Disease.

[27] Krinsky N.I., Deneke S.M. (1982). Interaction of oxygen and oxy-radicals with carotenoids. J. Natl. Cancer Inst..

[28] Burton G.W., Ingold K.U. (1984). beta-carotene: An unusual type of lipid antioxidant. Science.

[29] Miki W. (1991). Biological functions and activities of animal carotenoids. Pure Appl. Chem..

[30] Terao J. (1989). Antioxidant activity of beta-carotene-related carotenoids in solution. Lipids.

[31] Faria J.A.F., Mukai M.K. (1983). Use of gas chromatographic reactor to study lipid photoxidation. J. Am. Oil Chem. Soc..

[32] Handelman G.J., van Kuijk F.J.G.M., Chatterjee A., Krinsky N.I. (1991). Characterisation of products formed during the autoxidation of β-carotene. Free Radical Biol. Med..

[33] Epstein J.H. (1977). Effects of β-carotene on ultraviolet induced cancer formation in the hairless mouse skin. Photochem. Photobiol..

[34] Mathews-Roth M.M., Krinsky N.I. (1985). Carotenoid Dose Level and Protection against UV-B Induced Skin Tumors. Photochem. Photobiol..

[35] Santamaria L., Bianchi A., Mobilio G., Santagi G., Ravetto C., Bernardo G., Vetere C., Tryfiates G.P., Prasad K.N. (1988). Nutrition and Growth Cancer.

[36] Mathews-Roth M.M., Pathak M.A., Fitzpatrick T.B., Harber L.H., Kass E.H. (1977). Beta-carotene therapy for erythropoietic protoporphyria and other photosensitivity disease. Arch. Dermatol..

[37] Temple N.J., Basu T.K. (1988). Does beta-carotene prevent cancer? A critical appraisal. Nutr. Res..

[38] Lepage G. (1991). Federation of American Societies for Experimental Biology. 75th Annual Meeting. Atlanta, Georgia, April 21-25, 1991. Part II. Abstracts. FASEB J..

[39] Ames B.N. (1983). Dietary carcinogens and anti-carcinogens: Oxygen radicals and degenerative diseases. Science.

[40] Mathews-Roth M.M. (1982). Antitumor activity of β -carotene, canthaxanthin and phytoene. Oncology.

[41] Suda D., Schwartz J., Shklaret G. (1986). Inhibition of experimental oral carcinogenesis by topical beta carotene. Carcinogenesis.

[42] Som S., Chatterjee M., Banerjee M.R. (1984). β-Carotene inhibition of 7,12-dimethylbenz[a]anthracene-induced transformation of murine mammary cells in vitro. Carcinogenesis.

[43] Ramakrishnan T.V., Francis F.J. (1980). Autoxidation of carotenoids and their relative polarity. J. Food Qual..

[44] Karrer P., Helfenstein A., Wehrli H., Wettstein A. (1930). Pflanzenfarbstoffe XXV. Über die Konstitution des Lycopins und Carotins. Helv. Chim. Acta.

[45] Karrer P., Morf R., Schopp K. (1931). Zur kenntnis des vitamins-A aus fischtranen II. Helv. Chim. Acta..

[46] Hunter R.F., Williams N.E. (1945). Chemical conversion of β-carotene into vitamin-A. J. Chem. Soc..

[47] Glover J., Redfearn E.R. (1954). The mechanism of the transformation of beta-carotene into vitamin A. in vivo. Biochem. J..

[48] Zechmeister L., Le Rosen A.L., Schoeder W.A., Polhar A., Pauling L. (1943). Spectral characteristics and configuration of some stereoisomeric carotenoids including pro-lycopene and pro- β -carotene. J. Am. Chem. Soc..

[49] El-Tinay A.H., Chichester C. (1970). Oxidation of β -carotene. Site of initial attack, J. Org. Chem..

[50] Ganguly J., Sastry P.S. (1985). Mechanism of Conversion of β-Carotene into Vitamin A–Central Cleavage versus Random Cleavage. World Rev. Nutr. Diet..

[51] Mordi R.C., Walton J.C., Burton G.W., Hughes L., Ingold K.U., Lindsay D.A., Moffatt D.J. (1993). Oxidative Degradation of β-Carotene and β-Apo-8’-Carotenal. Tetrahedron.

[52] Mordi R.C., Walton J.C., Burton G.W., Hughes L., Ingold K.U., Lindsay D.A. (1991). Exploratory study of β-carotene autoxidation. Tetrahedron Lett..

[53] Kennedy T.A., Leibler D.C. (1991). Peroxyl radical oxidation of beta-carotene: Formation of beta-carotene epoxides. Chem. Res. Toxicol..

[54] Rodriguez E.B., Rodriguez-Amaya D.B. (2007). Formation of apo-carotenals and epoxy carotenoids from β -carotene by chemical reactions and by autoxidation in model systems and processed foods. Food Chem..

[55] Friend J. (1958). The coupled oxidation of β-carotene by a linoleate-lipoxidase system and by autoxidizing linoleate. Chem. Ind..

[56] Von Doering W.E., Sarma K. (1992). Stabilisation energy of polyethylene radicals: All *trans*-nonatetraenyl radical by thermal rearrangement of a semirigid{4-1-2}heptaene. Model of thermal stability of β-carotene. J. Am. Chem. Soc..

[57] Mohamed N., Hashim R., Rahman N.A., Zain S.M. (2001). An insight into the cleavage of β-carotene to vitamin A: A molecular mechanics study. J. Mol. Struct. (Thermo Chem).

[58] Penicaud C., Achir N., Dhuique-Mayer C., Dornier M., Bohuon P. (2011). Degradation of β-carotene during fruit and vegetable processing or storage: Reaction mechanisms and kinetic aspects: A review. Fruits.

[59] Achir N., Penicaud C., Avallone S., Bohuon P. (2011). Insight into β-carotene thermal degradation in oils with multiresponse modelling. J. Am. Oil Chem. Soc..

[60] Marx M., Stuparic M., Schieber A., Carle R. (2003). Effect of thermal processing on *cis*-*trans* isomerisation of β-carotene in carrot juices and carotene containing preparations. Food Chem..

[61] Henry L.K., Catignani G., Schwartz S. (1998). Oxidative degradation kinetics of lycopene, lutein and 9-*cis* and all-*trans*-β-carotene. J. Am. Oil Chem. Soc..

[62] Mordi R.C., Walton J.C. (2016). Identification of products from canthaxanthin oxidation. Food Chem..

[63] Møller A.H., Jahangiri A., Danielsen M., Madsen B., Joernsgaard B., Vaerbak S., Hammershøj M., Dalsgaard T.K. (2020). Mechanism behind the degradation of aqueous norbixin upon storage in light and dark environment. Food Chem..

[64] Mordi R.C. (1993). Mechanism of beta-carotene degradation. Biochem. J..

[65] Mayo F.R., Miller A.A. (1958). The oxidation of unsaturated compounds. VI. The effect of oxygen pressure on the oxidation of α-methylstyrene. J. Am. Chem. Soc..

[66] Porter N.A., Cudd M.A., Mller R.W., McPhail A.T. (1980). A fixed-geometry study of the S_H_2 reaction on the peroxide bond. J. Am. Chem. Soc..

[67] Porter N.A., Zuraw P.J. (1984). Stereochemistry of hydroperoxide cyclization reactions. J. Org. Chem..

[68] Bourgeois M.J., Maillard B., Montaudon E. (1986). Homolytic intramolecular displacements. 12. Decomposition of ethylenic peroxides: Effect of chain length. Tetrahedron.

[69] Mordi R.C., Walton J.C. (1990). Electron spin resonance study of free radicals generated from retinyl- and ionyl-derivatives. Chem. Phys. Lipids.

